# Detailed investigation of multiple resting cardiovascular parameters in relation to physical fitness

**DOI:** 10.1111/cpf.12800

**Published:** 2022-12-01

**Authors:** Lars Lind, Karl Michaëlsson

**Affiliations:** ^1^ Department of Medical Sciences Uppsala University Uppsala Sweden; ^2^ Department of Surgical Sciences Uppsala University Uppsala Sweden

**Keywords:** exercise test, physical fitness, pulse wave velocity, smoking, VO_2_‐max

## Abstract

**Objective:**

Maximal oxygen consumption at an exercise test (VO_2_‐max) is a commonly used marker of physical fitness. In the present study, we aimed to find independent clinical predictors of VO_2_‐max by use of multiple measurements of cardiac, respiratory and vascular variables collected while resting.

**Methods:**

In the Prospective study of Obesity, Energy and Metabolism (POEM), 420 subjects aged 50 years were investigated regarding endothelial function, arterial compliance, heart rate variability, arterial blood flow and atherosclerosis, left ventricular structure and function, lung function, multiple blood pressure measurements, lifestyle habits, body composition and in addition a maximal bicycle exercise test with gas exchange (VO_2_ and VCO_2_).

**Results:**

When VO_2_‐max (indexed for lean mass) was used as the dependent variable and the 84 hemodynamic or metabolic variables were used as independent variables in separate sex‐adjusted models, 15 variables showed associations with *p* < 0.00064 (Bonferroni‐adjusted). Eight independent variables explained 21% of the variance in VO_2_‐max. Current smoking and pulse wave velocity (PWV) were the two major determinants of VO_2_‐max (explaining each 7% and 3% of the variance; *p* < 0.0001 and *p* = 0.008, respectively). They were in order followed by vital capacity, fat mass, pulse pressure, and high‐density lipoprotein (HDL)‐cholesterol. The relationships were inverse for all these variables, except for vital capacity and HDL.

**Conclusion:**

Several metabolic, cardiac, respiratory and vascular variables measured at rest explained together with smoking 21% of the variation in VO_2_‐max in middle‐aged individuals. Of those variables, smoking and PWV were the most important.

## 1 INTRODUCTION

Maximal oxygen consumption at an exercise test (VO_2_‐max) is commonly used as a marker of physical fitness. VO_2_‐max has further been shown to be related to all‐cause mortality in a dose response fashion (Kodama, et al., 2009). VO_2_‐max is generally considered to have a strong genetic component and twin studies report heritability estimates of 0.5–0.7, although fitness is naturally also affected by lifestyle habits (Maes et al., 1996).

There are sex differences in VO_2_‐max, and VO_2_‐max declines with age (Amara et al., 2000; Serrano‐Sánchez et al., 2010) and increasing body fat (Serrano‐Sánchez et al., 2010). Other important determinants or consequences of low fitness are lung function at rest (forced vital capacity [FVC] and forced expiratory volume at 1 s [FEV1]) (Laukkanen et al., 2009; Mendelson et al., 2016; Nakamura et al., 2004) and smoking (Bernaards et al., 2003; de Borba et al., 2014; Suminski et al., 2009).

As reviewed by Rost (1997), cardiac enlargement in athletes was first described by Henshen in 1899 comparing cross‐country skiers with sedentary controls (Henschen, 1899). Later studies have also evaluated total heart size in physical fitness (Bouchard et al., 1977), but in most other studies, the heart size has been divided into left atrial (LA) size, left ventricular (LV) end‐diastolic diameter and LV mass using echocardiography to give more detailed information. All of these indices of heart size have been linked to cardiorespiratory fitness (Brinker et al., 2014; Gidding et al., 2010; Lam et al., 2010; Rogers et al., 2020).

Regarding other cardiovascular parameters, impaired endothelial vasodilatory capacity (Montero, 2015), increased aortic augmentation index (AIx) (Binder et al., 2006), increased arterial stiffness (Augustine et al., 2016; Boreham et al., 2004; Fernberg et al., 2017), poor LV diastolic function (Brinker et al., 2014), low haemoglobin level (Laukkanen et al., 2009) and carotid artery atherosclerosis (Rauramaa et al., 1995) have all been associated with poor VO_2_‐max.

A major disadvantage with previous studies is that they mainly have investigated a limited number of cardiovascular and lung function parameters in the same individuals. Accordingly, no comprehensive picture of the determinants of VO_2_‐max has been presented.

With the present study, we, therefore, aimed to measure multiple cardiovascular and lung function parameters in the same individuals and to relate those to VO_2_‐max. We used data from the population‐based Prospective study of Obesity, Energy and Metabolism (POEM), in which multiple cardiovascular and lung function parameters have been measured in the same individuals at the age of 50. We included all measured cardiovascular and lung function parameters in the analysis to capture as many facets of cardiorespiratory function as possible. The hypothesis tested was that we by this approach could explain a great proportion of the variance in VO_2_‐max.

## 2 METHODS

In a population‐based study of individuals, all aged 50 years, in Uppsala City, Sweden, the POEM (Lind, 2013), a random sample of men and women were invited to participate 1 month following their 50th birthday. The inclusion in the study started in 2012 and was stopped in 2017. The participation rate was 25%, and the inclusion was stopped after 502 participants. The study was approved by the Ethics Committee of the University of Uppsala, and the subjects gave their written informed consent to participate.

The participants were asked how many times a week they performed mild (such as walking) and harder (to produce perspiration, like running) exercises for at least 30 min. Based on these data, four groups were defined (see Lind et al., 2021 for details): sedentary (13% of the sample), mild exercise only (24%), some harder exercise (33%) and harder exercise (30%).

All individuals were investigated in the morning after an overnight fast. An arterial cannula was inserted in the brachial artery for blood sampling and was later used for regional infusions of vasodilators. Lipid variables and fasting blood glucose were measured by standard laboratory techniques. Height was recorded by a ruler and body weight was measured on a scale (Tanita BC‐418). Thereafter, multiple physiological tests were performed.

Endothelial function and arterial compliance/stiffness were both measured by three different techniques: acetylcholine‐mediated increase in forearm blood flow, flow‐mediated vasodilation (FMD) and peripheral artery vasodilation (EndoPath). The carotid arteries were investigated by ultrasound for anatomy (intima‐media thickness and echolucency and blood flow. The myocardial LV was evaluated by ultrasound for LV geometry (LV mass, end‐diastolic volume, wall thickness), systolic (ejection fraction) and diastolic function (isovolumetric relaxation time, E/A‐ratio, Doppler e′/a′ ratio). Blood pressure was measured by four different techniques (conventional, invasive, derived central pressure, 24 h ambulatory). Basal energy expenditure was measured by indirect calorimetry and heart rate variability (HRV) was recorded for 5 min. Arterial compliance was measured by three techniques (carotid‐femoral pulse wave velocity [PWV], carotid artery distensibility and the stroke volume to pulse pressure ratio). Radial artery pulse wave was recorded for the AIx and reflectance index. Blood flow of the brachial artery was recorded at rest and following 5 min of hyperaemia.

Total and regional body fat and lean mass were estimated using dual‐energy X‐ray absorptiometry (DXA; Lunar Prodigy, GE Healthcare). To minimize the potential operator bias, all scans were performed in the same room by one experienced nurse. Total fat and lean mass had a precision error of 1.5% and 1.0%, respectively. For analysis, automatic edge detection was always used; however, all scans were thoroughly checked for errors and manually corrected if needed.

On a separate day, close to the first investigations, the participants returned to the nonfasted state to evaluate lung function (FVC and FEV1) and to perform a maximal bicycle ergometer test with gas exchange recordings. Also, the recoveries of the heart rate, blood pressure and VO_2_ and VCO_2_ during 5 min were recorded.

Smoking was identified as current smoking.

All the physical investigations have previously been described by Lind and Lampa (2019) and are given in detail in the Supporting Information.

### 2.1 Statistical analysis

All variables were checked for a normal distribution, and some variables such as the E/A ratio, serum triglycerides, most HRV variables, were skewed to the right and therefore ln‐transformed to achieve a normal distribution to be used in the models.

First, the relationship between VO_2_‐max and sex was investigated by ANOVA (same age of all subjects). Second, the relationships between VO_2_‐max (adjusted for lean mass) and the 84 hemodynamic or metabolic variables were investigated one by one in sex‐adjusted linear regression models. Third, the relationships between VO_2_‐max and the 84 hemodynamic or metabolic variables were investigated one by one with sex and fat mass included in the model. Fourth, the interactions between sex and the hemodynamic or metabolic variables were investigated one by one. Fifth, a multiple linear model with VO_2_‐max as the outcome and sex together with eight other hemodynamic or metabolic variables, which were Bonferroni‐significant in the initial analyses, as independent variables were evaluated. In this model, variables being closely related (correlation coefficient > 0.3) to other more significant variables, such as FEV1, and several blood pressure and heart rate measurements, were not included in this multiple model due to the risk of co‐linearity. In the second to fourth steps, the relationships between VO_2_‐max and the 84 hemodynamic or metabolic variables were investigated one by one, and therefore, Bonferroni adjustment for these tests was performed resulting in a critical *p*‐value of 0.00064. In step five, we regarded *p* < 0.05 to be significant.

STATA14 was used for the calculations (Stata Inc.).

## 3 RESULTS

The median and interquartile ranges of measured variables are given in Supporting Information: Table 1.

VO_2_‐max (alone and when adjusted for lean mass) was a normally distributed variable. The mean for unadjusted VO_2_‐max was 2.79 (SD 0.54) L/min in men and 1.85 (0.40) in women (*p* < 0.0001). Sex explained 49% of the variation in unadjusted VO_2_‐max. VO_2_‐max adjusted for lean mass was 0.46 (SD 0.079) L/min/kg lean mass in men and 0.39 (0.083) in women (*p* = 0.0043). Sex explained only 1.7% of the variation in VO_2_‐max after adjustment for lean mass. In the following analysis, VO_2_‐max adjusted for lean mass was used. Current smoking was reported by 9.8% of the individuals.

**Figure 1:**
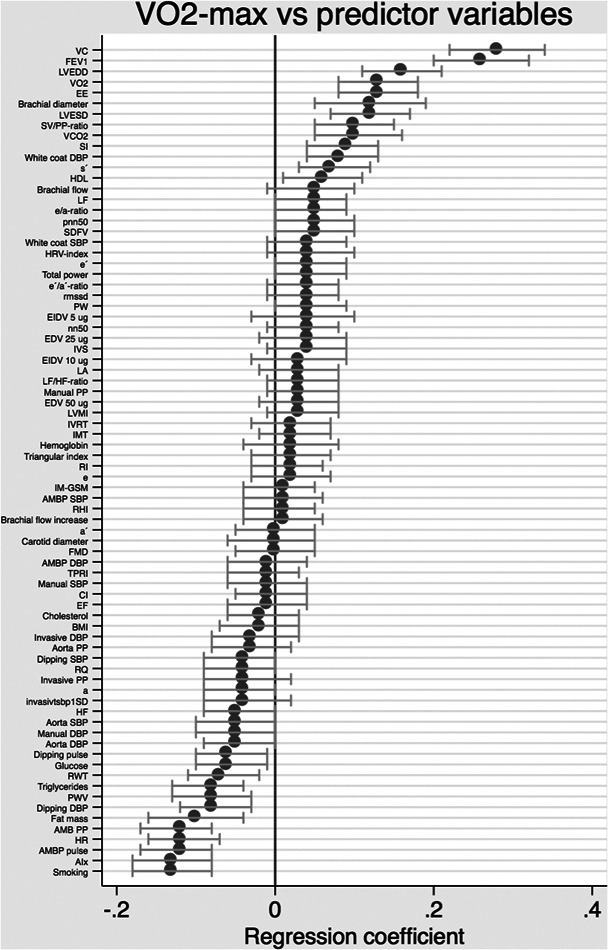
Relationships between hemodynamic and metabolic variables and VO_2_‐max (adjusted for lean mass) when the hemodynamic and metabolic variables were evaluated one by one. The regression coefficient and 95% CIs are given for the sex‐adjusted analyses. a, atrial contraction transmitral filling velocity; AIx, aortic augmentation index; AMBP, ambulatory monitoring of blood pressure; BMI, body mass index; CI, cardiac index; DBP, diastolic blood pressure; e, early transmitral filling velocity; EDV, endothelium‐dependent vasodilatation; EE, energy expenditure; EF, ejection fraction; EIDV, endothelium‐independent vasodilatation; FEV1, forced expiratory volume at 1 s; FMD, flow‐mediated dilatation; HDL, high‐density lipoprotein; HR, heart rate; HRV, heart rate variability; IM‐GSM, echogenicity of the intima‐media complex; IMT, intima‐media thickness; IVRT, isovolumetric relaxation time; IVS, intraventricular thickness; LA, left atrial diameter; LF, low frequency; LF/HF ratio, low‐frequency/high‐frequency ratio; LVEDD, left ventricular end‐diastolic diameter; LVESD, left ventricular end‐systolic diameter; LVMI, left ventricular mass index; PP, pulse pressure; PW, posterior wall thickness; PWV, pulse wave velocity; RHI, reactive hyperaemia index; RI, reflectance index; RQ, respiratory quote; RWT, relative wall thickness; SBP, systolic blood pressure; SDFV, systolic to diastolic blood flow velocity; SI, stroke index; SV/PP‐ratio, stroke volume to pulse pressure ratio; TPRI, total peripheral resistance index; VC, Vital capacity; VCO_2_, carbon dioxin production; VO_2_, oxygen consumption.

When VO_2_‐max was used as the dependent variable and the 84 hemodynamic or metabolic variables were used as independent variables in separate sex‐adjusted models for each hemodynamic or metabolic variable, 15 variables showed associations with *p* < 0.00064 (Bonferroni adjusted threshold, see Supporting Information: Table 2 and Figure 1 for details). Vital capacity, FEV1 and high‐density lipoprotein (HDL) were positively related to VO_2_‐max, while the pulse rate, pulse pressure, diastolic night‐time dipping at 24 h ambulatory recording, BMI, fat mass, triglycerides, office recordings of the pulse rate, diastolic blood pressure and calculated central systolic and diastolic blood pressure all were related to VO_2_‐max in a negative fashion. All of these variables displayed *p* < 0.05 when additional adjustment for fat mass was made. No interaction term between sex and any hemodynamic or metabolic variable was significant following adjustment for multiple testing.

Together with smoking, eight hemodynamic or metabolic variables being Bonferroni‐significant in the initial analyses explained 21% of the variation in VO_2_‐max. This held true also after omitting the variable sex, which was included in the first version of the model.

**Figure 2:**
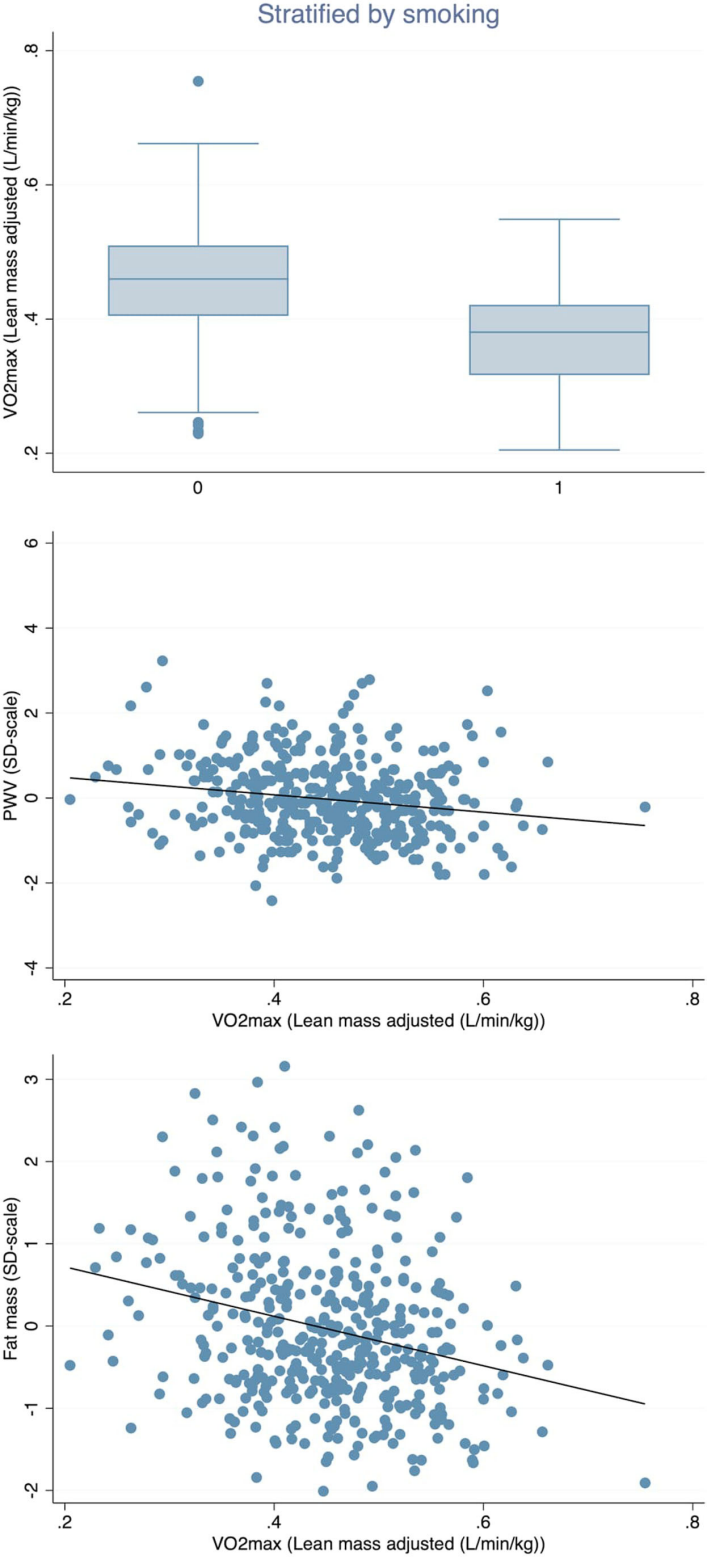
Relationships between VO_2_‐max (adjusted for lean mass) and variables were found to be of major importance to explain the variation in VO_2_‐max (adjusted for lean mass). VO_2_‐max versus current smoking is given in the upper panel. VO_2_‐max versus pulse wave velocity (PWV) is in the middle panel and VO_2_‐max versus fat mass is given in the lower panel.

In this model with VO_2_‐max as the outcome, smoking and PWV were the two major determinants of VO_2_‐max (explaining 7%, *p* < 0.0001 and explaining 3%, *p* = 0.008, respectively). They were followed by vital capacity, fat mass, pulse pressure and HDL‐cholesterol, which all showed *p* < 0.05 in this multiple model (see Table 1 for details). The relationships were inverse for all these variables, except for vital capacity and HDL. Sex (*p* = 0.97), triglycerides and the resting heart rate showed *p* > 0.05. Figure 2 displays some of these main relationships more in detail.

**Table 1:** Relationships between VO_2_‐max (outcome, adjusted for lean mass) and sex and eight hemodynamic or metabolic variables as independent variables

Variables related to VO_2_‐max	Beta	95% CI low	95% CI high	*p* Value
Sex	−0.004335	−0.2405681	0.2318981	0.971
Ambulatory pulse pressure	−0.1310065	−0.2354139	−0.0265991	0.014
Smoking	−0.2153444	−0.2965848	−0.1341041	0.000
Fat mass	−0.1095866	−0.1923139	−0.0268592	0.010
Resting heart rate	−0.0134249	−0.1198909	0.093041	0.804
Triglycerides	−0.0539096	−0.1287491	0.02093	0.158
Pulse wave velocity	−0.112147	−0.1944053	−0.0298887	0.008
HDL	0.0920998	0.0008397	0.1833599	0.048
Resting vital capacity	0.1385489	0.0300567	0.2470411	0.012

Abbreviations: CI, cardiac index; HDL, high‐density lipoprotein; VO_2_‐max, maximal oxygen consumption.

## 4 DISCUSSION

The present study showed that smoking and an increased PWV at rest were most closely related to VO_2_‐max, but lung function, fat mass, pulse pressure and HDL‐cholesterol were also related to this commonly used marker of physical fitness.

### 4.1 Comparison with the literature

All of these variables have previously been shown to be related to VO_2_‐max, as cited in the Introduction section. The novelty of the present study is that we by the measurements of multiple cardiovascular and lung function variables in the same individuals were able to compare these variables in terms of importance and independence from each other.

We could not reproduce some other previous findings that endothelial vasodilatory function (FMD) (Montero, 2015), a poor LV diastolic function and a large LV end‐diastolic volume (Brinker et al., 2014), a low haemoglobin level (Laukkanen et al., 2009) and carotid artery atherosclerosis (Rauramaa et al., 1995) were related to VO_2_‐max.

One major advantage of the present study is that we could evaluate the independent contribution of indices reflecting different aspects of physiology in the same model and found that several different physiological pathways are determinants of VO_2_‐max. This is not a surprise, since it is obvious that the heart, the lungs and the skeletal muscles simultaneously all play important roles in the determination of cardiorespiratory fitness.

Given that basic assumption, it was a surprise that no variable reflecting myocardial function or structure was amongst the major identified physiological indices. One explanation for this could be the very strict Bonferroni adjustment applied to compensate for the multiple statistical testing. It could be seen that both the s′ and e′ at TDI, the e′/a′‐ratio at TDI, stroke index, LA diameter (inverse) and relative wall thickness (RWT) (inverse) showed *p* < 0.05 (*p* = 0.054 for RWT). Thus, if not using this strict adjustment for multiple testing, we could replicate the findings of others that several myocardial indices are linked to VO_2_‐max, although other factors might be more important.

Only a small part of the variance in VO_2_‐max could be explained by the evaluated variables despite that a great number of cardiopulmonary variables were assessed. Several factors could explain this finding. First, all variables have a certain lack of precision and variability in measurements that could lower the degree of explained variance, especially when several variables seem to be of importance. Second, all variables were measured at rest. It could be speculated that a better R2 for VO_2_‐max would be obtained if the variables were measured during exercise instead. Third, certain factors of particular interest were not measured. One such very important feature is the mitochondrial function in the heart and skeletal muscles during exercise. Another could be diffusion capacity in the lungs. Yet another factor is skeletal muscle composition, which is important for endurance capacity (Hall et al., 2021). Fourth, it has been shown that genetic DNA variations both at the global level (Gineviciene et al., 2022), as well as at the mitochondrial level (Vellers et al., 2020), are important determinants of VO_2_‐max. Fifth, we normalized VO_2_‐max for lean mass measured at DXA. Most other studies have not performed such rigorous normalization, and if no normalization would have been performed, lean mass in itself would explain 60% of the variance in VO_2_‐max.

### 4.2 Clinical perspectives

Apart from an increase in endurance training, smoking cessation would be the single most important action to improve VO_2_‐max, as suggested by the present findings. We could not however find any intervention studies to support that assumption.

It might also be warranted to reduce arterial stiffness, although the causality is less clear in this case. In a small placebo‐controlled trial in postmyocardial infarction patients, treatment with a combination of a statin and an angiotensin‐receptor blocker reduced PWV (Turk Veselič et al., 2018). In an open trial of the combination of an ACE inhibitor and a calcium channel blocker in patients with hypertension, an improvement in PWV was seen after 12 months (Radchenko et al., 2018). It would be of interest to see if such interventions that improve arterial stiffness would also have an impact on VO_2_‐max.

Weight loss might also be a way to increase VO_2_‐max, and at least in patients with class III obesity (BMI > 40 kg/m^2^), weight reduction increased VO_2_‐max (Hakala et al., 1996).

### 4.3 Strengths and limitations

The major strength of the present study is the multitude of cardiovascular and lung function variables measured at rest together with VO_2_‐max in individuals of the same age. Since age is an important determinant of VO_2_‐max (Amara et al., 2000; Serrano‐Sánchez et al., 2010), standardization of age would remove the impact of this very important variable on the variance in VO_2_‐max. Another strength is that we could adjust VO_2_‐max for lean mass, measured by the gold standard, DXA. As could be seen in our analysis, this standardization removed most of the sex effect on the variation in VO_2_‐max.

This is a cross‐sectional study, and as such causality can never be proven and the directions of relationships are not clear.

A limitation of studying a homogeneous sample is that the generalizability is low, so the present results have to be reproduced in samples from other countries with other ethnical groups, as well as in other age groups.

## 5 CONCLUSION

Several metabolic, cardiac, respiratory and vascular variables measured at rest explained together with smoking 21% of the variation in VO_2_‐max in individuals aged 50 years.

## CONFLICT OF INTEREST

The authors declare no conflict of interest.

## Supporting information

Supporting information.

## Data Availability

According to Swedish law, personal health data cannot be made publicly available. Data from this study are however available upon a reasonable request by other researchers.
