# Supplementary material for: Detailed investigation of multiple resting cardiovascular parameters in relation to physical fitness
Source: Clin Physiol Funct Imaging. 2022 Dec 1;43(2):120–7. doi: 10.1111/cpf.12800 (PMC10108008; doi:10.1111/cpf.12800)
Supplement: Supplementary file 1 — Supporting information. [file CPF-43-120-s001.docx]

### Supplementary information for:

# A DETAILED INVESTIGATION OF MULTIPLE RESTING CARDIOVASCULAR PARAMETERS IN RELATION TO PHYSICAL FITNESS

Lars Lind^1^ and Karl Michaëlsson^2^

Departments of Medical^1^ and Surgical Sciences^2^, Uppsala University, Uppsala, Sweden

### DETAILED METHODS DESCRIPTION

### Bicycle exercise test with gas exchange

Using a bicycle ergonometer, a maximal exercise test with gas exchange measurements was performed (Jaeger Oxygen Pro, Vyaire medical, Mattawa, IL, USA). Blood pressure, heart rate, vital capacity, VO_2_ and VCO_2_ were measured at rest and thereafter the participants were asked to work until exhaustion. The work load was increased by 10W/min, starting at 30W for women and 50W for men. The maximal VO_2_ during the last minute of work was recorded.

### Blood pressure

Blood pressure was manually measured by a mercury sphygmomanometer in the supine position after 30 min of rest. By applying radial pulse wave recordings (see below), central blood pressure was calculated by the software included in the commercial device. Blood pressure was also measured invasively in the brachial artery (see below) by an electronic transducer in the supine position after 30 min of rest, as well as by an automatic device using the ocillometric technique (Omron HEM-711, Kyoto, Japan). 24h ambulatory blood pressure (AMBP) was measured every 30 min during the awake period and every 60 min during night-time (Meditech ABPM-5, Budapest, Hungary). According to the patient history of the sleeping period, the night/day pressure ratio was calculated. The white-coat effect was defined as the manual BP measurement minus the day-time AMBP measurement.

### The invasive forearm technique

Forearm blood flow was measured by venous occlusion plethysmography (Elektromedicin, Kullavik, Sweden). After evaluation of resting forearm blood flow, local intra-arterial drug-infusions were given in the brachial artery during 5 minutes for each dose. At the end of each infusion step, forearm blood flow was evaluated. The infused dosages were 25 and 50 ug/minute for Acetylcholine (Clin-Alpha, Basel, Switzerland) to evaluate Endothelium-dependent vasodilation (EDV) in forearm resistance vessels and 5 and 10 ug/minute for SNP (Nitropress, Abbot, UK) to evaluate endothelium-independent vasodilation (EIDV). The two drugs were given in a random order between subjects.

Endothelium-dependent vasodilation in forearm resistance vessels was defined as forearm blood flow during infusion of either 25 or 50 ug/min of Acetylcholine minus resting forearm blood flow divided by resting forearm blood flow (denoted EDV 25 ug and EDV 50 ug). Endothelium-independent vasodilation in forearm resistance vessels was defined as forearm blood flow during infusion of either 5 or 10 ug/min of SNP minus resting forearm blood flow divided by resting forearm blood flow (denoted EIDV 5 ug or EIDV 10 ug).

### The brachial artery ultrasound technique

The brachial artery diameter was assessed by external B-mode ultrasound imaging 2 – 3 cm above the elbow (Acuson XP128 with a 10 MHz linear transducer, Acuson Mountain View, California, USA). Following measurement of the brachial artery diameter at rest, a blood flow increase was induced by inflation of a pneumatic cuff placed around the forearm to a pressure at least 50 mmHg above systolic blood pressure for 5 min and then a suddenly release of the cuff. Flow-mediated vasodilation (FMD) was defined as the maximal brachial artery diameter recorded between 30 and 90 seconds following cuff release minus diameter at rest divided by the diameter at rest. Blood flow velocity was recorded by Doppler at rest and in the very early hyperaemic period (within5-10 seconds). From the Doppler velocity time integral (VTI) recording, heart rate and the diameter of the vessel, the blood flow was calculated (in ml/min). The blood flow increase during the induced hyperaemia was defined as blood flow during hyperaemia minus resting blood flow divided by resting blood flow. The mean blood flow velocity during the early hyperaemic period was also calculated during the systolic and diastolic phases separately, and the systolic to diastolic blood flow velocity ratio (SDFV) was calculated.

### Peripheral artery tonometry

Peripheral artery tonometry (PAT) was performed using the EndoPat commercial devise (Itamar, Jerusalem, Israel). By applying finger-tip sensors, the blood pressure amplitude was measured at rest in one fingertip in each hand before and then continuously during 5 min after 5 min of arterial occlusion of one of the forearms by inflation of a pneumatic cuff placed around the forearm to a pressure at least 50 mmHg above systolic blood pressure. From these recordings, software included in the device calculates the reactive hyperaemia index (RHI, see for more details: <http://www.itamar-medical.com/What_is_EndoPAT.html)>. A high RHI is regarded as an index of a good peripheral endothelial function.

### Pulse wave analysis

A micromanometer tipped probe (Sphygmocor, Pulse Wave Medical Ltd, Sydney, Australia) was applied to the surface of the skin overlying the radial artery and the peripheral radial pulse wave was continuously recorded. The mean values of around 10 pulse waves were used for analyses. Based on transfer functions included in the device, aortic systolic and diastolic blood pressures were calculated from the radial recordings. Also the augmentation index (AIx), the ratio between the first reflected wave and the systolic peak, and the reflectance index (RI), the ratio between the second reflected wave and the systolic peak were calculated. (see Lind et al (Lind*, et al.* 2006) for details on definitions).

The pulse wave curve was also recorded by aplanation tonometry using the Sphygmocor device in the carotid artery and the femoral artery in parallel with an ECG recording in order to calculate the time difference of the appearance of the systolic elevation in blood pressure. The distance from the carotid artery to the femoral artery was measured and when divided by the time difference of the appearance of the systolic elevation in blood pressure in the carotid and femoral arteries, the aortic pulse wave velocity is obtained.

### Carotid artery compliance

The diameter of the common carotid artery of the right side 1-2 cm proximal of the bifurcation was measured by ultrasound M-mode at its maximal diameter in systole and the minimal diameter in diastole (Acuson XP128 with a 10 MHz linear transducer, Mountain View, California, USA). The distensibility of the carotid artery was calculated as the change in diameter maximum to minimum in relation to the minimal diameter in diastole divided by the central pulse pressure obtained by pulse wave analysis. Stiffness beta was calculated as the ratio of the natural logarithm of SBP/DBP to the relative change in diameter, while Young´s elastic modulus was given by the formula: (pulse pressure*CCA diastolic diameter)/change in diameter*vessel thickness).

### Stroke volume to pulse pressure ratio

Echocardiography was performed (2.5 MHz transducer, Acuson XP124, Mountain View, California, USA) and using the Teichholz formula at the M-mode recording in the parasternal recording, ejection fraction and stroke volume were calculated. The stroke volume to pulse pressure (SV/PP) ratio was calculated as stroke volume divided by central pulse pressure (achieved by pulse wave analysis).

### Carotid artery ultrasound evaluation

The carotid artery was assessed by external B-mode ultrasound imaging (Acuson XP128 with a 10 MHz linear transducer, Mountain View, California, USA). The intima-media thickness (IMT) was evaluated in the far wall in the common carotid artery (CCA) 1-2 cm proximal to the bulb.

The images were digitised and imported into the AMS (Artery Measurement Software, Gothenburg, Sweden) automated software for dedicated analysis of IMT and the grey scale median of the intima-media complex. A 10 mm segment with good image quality was chosen for IMT-analysis from the carotid artery. The value obtained is the mean of around 100 discrete measurements over the 10 mm segment. The given value for carotid artery intima-media thickness is the mean value from both sides.

A region of interest was placed manually around the intima-media segment that was evaluated for IMT and the programme calculates the echogenicity (grey scale) of the intima-media complex from analysis of the individual pixels within the region of interest on a scale from 0 (black) to 256 (white). The blood was used as the reference for black and the adventitia was the reference for white. The GSM-value given is the mean value from both sides.

Blood flow velocity was recorded by Doppler at rest in the CCA. From the Doppler velocity time integral (VTI) recording, heart rate and the diameter of the vessel, the blood flow was calculated (in ml/min). The mean blood flow velocity during the early hyperaemic period was also calculated during the systolic and diastolic phases separately, and the systolic to diastolic blood flow velocity ratio (SDFV) was calculated.

### Echocardiography and Doppler

A comprehensive two-dimensional and Doppler echocardiography was performed with an Acuson XP124 cardiac ultrasound unit (Acuson, California, USA). A 2.5 MHz transducer was used for the majority of the examinations.

LV dimensions were measured with M-mode on-line from the parasternal projection~~s~~, using a leading-edge to leading-edge convention. Measurements included left atrial diameter (LA), interventricular septal thickness (IVS), posterior wall thickness (PW), left ventricular diameter in end diastole and end systole (LVEDD, LVESD). Left ventricular relative wall thickness (RWT) was calculated as (IVS+PW)/LVEDD.

Left ventricular mass (LVM) was determined from the Penn conversion. LVM was then indexed for height^2.7^ to obtain left ventricular mass index (LVMI).

Left ventricular volumes were calculated according to the Teichholz formula (7*D3/(2.4+D) and from that value stroke index (SI) and ejection fraction (EF) were calculated.

The left ventricular diastolic filling pattern of the mitral inflow was obtained from the apical transducer position with the pulsed Doppler sample volume between the tips of the mitral leaflets during diastole. The peak velocity of the early rapid filling wave (E wave) and the peak velocity of atrial filling (A wave) were recorded and the E to A ratio (E/A) was calculated. Left ventricular isovolumic relaxation time (IVRT) was measured as the time between aortic valve closure and the start of mitral flow using the Doppler signal from the area between the left ventricular outflow tract and mitral flow.

Using tissue Doppler imaging (DTI) with the sampling volume in the proximal part of the IVS, the maximal systolic (s´), early diastolic (e´) and atrial-induced (a´) velocities of the motion of the IVS were recorded. Similar to the transmitral E/A-ratio, the e´/a´-ratio was calculated as well as the E/e´-ratio.

Cardiac index (CI) was calculated from SI and HR. Total peripheral resistance index (TPRI) was calculated as mean arterial blood pressure minus 3 divided by CI.

### Indirect calorimetry

In the fasting state and supine position at rest, metabolic gas exchange was measured during 30 min in a ventilated hood (Jaeger Oxygen Pro, Vyaire medical, Mattawa, IL, USA) by measurements of the lowering of the oxygen tension and increase in carbon dioxide tension and the airflow through the hood. From the oxygen consumption (VO2) and carbon dioxide production (VCO2) measurements, the respiratory quotient (RQ) and basal energy expenditure (EE) were calculated.

### Heart rate variability

Heart rate variability (HRV) was measured during 5 min of rest in the supine position by a commercial device (Sphygmocor, Pulse Wave Medical Ltd, Sydney, Australia). From this 5 min recording calculations were performed by the software included in the device regarding indices reflecting HRV in both the time and frequency domains. SDNN (ms) is the standard deviation of the all NN interval. pNN50 (%) is the proportion of RR intervals having a difference of >50 msec. Triangular Index (ms) is the integral of the density distribution (ie number of all NN intervals plotted in a histogram) divided by the maximum of the density distribution. RMSSD (m/s) is the square root of the mean squared differences of successive NN intervals. The low (0.05-0.15 Hz) and high (>0.15 Hz) frequency bands are given as both maximal values and normalized values and their ratio.

### References

Lind L, Fors N, Hall J, Marttala K and Stenborg A. A comparison of three different methods to determine arterial compliance in the elderly: the Prospective Investigation of the Vasculature in Uppsala Seniors (PIVUS) study. *J Hypertens* (2006); **24**: 1075-1082.

### Supplementary Table 1

Median and interquartile range (IQR) for the studied variables.

| **Variable** | **n** | **Median (IQR)** |
| --- | --- | --- |
| Fat mass (kg) | 426 | 22.6 (17.8, 29.8) |
| Fasting blood glucose (mmol/l) | 426 | 4.5 (4.2, 4.8) |
| Serum cholesterol (mmol/l) | 426 | 5.3 (4.7, 5.9) |
| HDL-cholesterol (mmol/l) | 426 | 1.3 (1.1, 1.6) |
| Serum triglycerides (mmol/l) | 426 | 1 (.7, 1.4) |
| Hemoglobin (g/l) | 426 | 139 (130, 145) |
| BMI (kg/m^2^) | 426 | 25.7 (23.4, 28.5) |
| Invasive SBP (mmHg) | 321 | 124 (115, 135) |
| Invasive DBP (mmHg) | 321 | 70 (65, 76) |
| Invasive PP (mmHg) | 321 | 54 (48, 60) |
| Manual SBP (mmHg) | 426 | 124 (114, 134) |
| Manual DBP (mmHg) | 426 | 76 (70, 82) |
| Manual PP (mmHg) | 426 | 48 (42, 54) |
| Central SBP (mmHg) | 424 | 115 (106, 125.5) |
| Central DBP (mmHg) | 424 | 78 (71, 83) |
| Central PP (mmHg) | 424 | 38 (32, 44) |
| Ambulatory SBP (mmHg) | 416 | 120 (112, 129) |
| Ambulatory DBP (mmHg) | 416 | 73 (68, 79.5) |
| Ambulatory PP (mmHg) | 416 | 70 (65, 77) |
| Night/day-ratio SBP | 413 | .9 (.8, .9) |
| Night/day-ratio DBP | 413 | .8 (.8, .9) |
| Night/day-ratio heart rate | 413 | .9 (.8, .9) |
| White coat effect SBP (mmHg) | 416 | 1 (1, 1.1) |
| White coat effect DBP (mmHg) | 416 | 1 (1, 1.1) |
| Invasive HR (beats/min) | 426 | 55 (50, 61) |
| Ambulatory HR (beats/min) | 416 | 70 (65, 77) |
| LA (mm) | 415 | 37 (35, 40) |
| IVS (mm) | 406 | 8 (8, 9) |
| LVEDD (mm) | 406 | 53 (49, 56) |
| PW (mm) | 406 | 8 (8, 8) |
| LVESD (mm) | 406 | 30 (27, 32) |
| Transmitral E-wave (cm/s) | 411 | 56 (48, 65) |
| Transmitral A-wave (cm/s) | 411 | 37 (31, 44) |
| IVRT (ms) | 400 | 87 (78, 97) |
| s´ at TDI (cm/s) | 409 | 7.3 (6.9, 7.8) |
| e´ at TDI (cm/s) | 409 | 10.3 (9.1, 11.6) |
| a´ at TDI (cm/s) | 408 | 9.2 (8.3, 10.6) |
| RWT | 406 | .3 (.3, .3) |
| e/a-ratio at TDI | 411 | 1.5 (1.3, 1.7) |
| e´/a´-ratio at TDI | 408 | 1.1 (.9, 1.3) |
| E/e´-ratio at TDI | 405 | 5.5 (4.7, 6.3) |
| LVMI (g/m^2.7^) | 406 | 34.5 (30.2, 39.8) |
| LVEDV (cm^3^) | 406 | 135.3 (112.8, 153.7) |
| LVESV (cm^3^) | 406 | 35 (27, 41) |
| EF | 406 | .7 (.7, .8) |
| SI (ml/BSA) | 406 | 50.7 (44.3, 58.8) |
| CI (L/min/BSA) | 406 | 2.8 (2.4, 3.3) |
| TPRI (dynes*s*cm^-5^*BSA) | 406 | 2540.2 (2165.6, 3016.2) |
| Resting VO_2_ (ml/min) | 410 | 201 (175, 227) |
| Resting VCO_2_ (ml/min) | 410 | 156 (135, 178) |
| Resting RQ | 410 | .8 (.8, .8) |
| Resting energy expenditure (kcal) | 409 | 1355 (1183, 1541) |
| Resting VC (L) | 426 | 4.4 (3.7, 5.2) |
| FEV1 (L) | 425 | 3.5 (3, 4.1) |
| RMSSD (ms) | 415 | 33.4 (24.1, 47.9) |
| HRV-index (ms) | 415 | 9.1 (7.3, 11.3) |
| pNN50 (%) | 389 | 10.5 (4.1, 21.9) |
| NN50 (ms) | 389 | 32 (13, 66) |
| Triangular index (ms) | 415 | 297 (226, 392) |
| LF maximum (Hz) | 415 | .1 (.1, .1) |
| HF maximum (Hz) | 415 | .2 (.2, .2) |
| LF/HF ratio | 415 | 1.5 (.8, 2.9) |
| Total power (ms^2^) | 414 | 1477.5 (857, 2432) |
| LF (normalized) (%) | 414 | 60.8 (45, 74.1) |
| HF (normalized) (%) | 414 | 39.4 (26.3, 55.1) |
| IMT (mm) | 421 | .6 (.6, .7) |
| IM-GSM | 421 | 66.4 (55.8, 77.6) |
| Carotid diameter (mm) | 421 | 5.9 (5.5, 6.3) |
| SV/PP-ratio (ml/mmHg) | 404 | 2.6 (2.1, 3.2) |
| AIx | 424 | 139 (126.5, 152) |
| EDV 25 µg (%) | 318 | 368.1 (198.8, 570.7) |
| EDV 50 µg (%) | 318 | 513.2 (295.4, 774.5) |
| EIDV 5 µg (%) | 203 | 245.9 (141.6, 389.9) |
| EIDV 10 µg (%) | 203 | 362.6 (226.4, 530.1) |
| Brachial diameter (mm) | 401 | 3.5 (3, 4) |
| Brachial flow at rest  (ml/min/100 ml tissue) | 398 | 3985.3 (2904.4, 5596.9) |
| FMD (%) | 393 | 6.4 (2.8, 9.1) |
| Brachial flow increase (%) | 376 | 8.9 (6.9, 11.1) |
| PWV (m/s) | 416 | 6.9 (6.3, 7.6) |
| SDFV ratio | 379 | 1.6 (1.4, 1.8) |
| RI | 424 | 50 (44, 55) |
| RHI | 411 | 2.3 (2, 2.7) |

HDL=High density lipoprotein, BMI=Body mass index, SBP=Systolic blood pressure, DBP=Diastolic blood pressure, PP=Pulse pressure, HR=Heart rate, LA=Left atrial diameter, IVS=Intraventricular thickness, LVEDD=Left ventricular end-diastolic diameter, PW=Posterior wall thickness, LVESD=Left ventricular end-systolic diameter, E=Early transmitral filling velocity, A=Atrial contraction transmitral filling velocity, IVRT=Isovolumetric relaxation time, TDI=Tissue Doppler imaging, RWT=Relative Wall thickness, LVMI=Left ventricular mass index, LVEDV=Left ventricular end-diastolic volume, LVESV=Left ventricular end-systolic volume, EF= Ejection fraction, SI= Stroke index, BSA=Body surface area, CI=Cardiac index, TPRI=Total peripheral resistance index, VO_2_=Oxygen consumption, VCO_2_=Carbon dioxin production, RQ=Respiratory quote, VC=Vital capacity, FEV1=Forced expiratory volume at 1 s, HRV=Heart rate variability, LF=Low frequency, HF=High frequency, IMT=Intima-media thickness, IM-GSM=Echogenicity of the intima-media complex, SV/PP-ratio=Stroke volume to pulse pressure ratio, AIx=Aortic augmentation index, EDV=Endothelium-dependent vasodilatation, EIDV=Endothelium-independent vasodilatation, FMD=Flow-mediated dilatation, PWV=Pulse wave velocity, SDFV ratio=Systolic to diastolic blood flow velocity ratio, RI=Reflectance index, RHI=Reactive hyperemia index

### Supplementary Table 2

Relationships between hemodynamic and metabolic variables and VO_2_-max (adjusted for lean mass) when the hemodynamic and metabolic variables were evaluated one by one. Beta, 95% CI and p-value are given for the sex-adjusted analyses. P-values are also given for further adjustment for fat mass and for the interaction between sex and the hemodynamic and metabolic variables. P< 0.00064 is the Bonferroni adjusted level of significance. The table is sorted according to the sex-adjusted p-value.

| **Variable** | **Beta** | **95% CI low** | **95% CI high** | **Sex adjusted p-value** | **Fat mass-adjusted p-value** | **Sex- interaction p-value** |
| --- | --- | --- | --- | --- | --- | --- |
| Ambulatory PP (mmHg) | -.22 | -.3 | -.15 | 3.02e-08 | 2.64e-06 | .5599314 |
| Smoking | -.23 | -.32 | -.15 | 6.99e-08 | 1.23e-08 | .215923 |
| Fat mass (kg) | -.2 | -.28 | -.12 | 5.18e-07 | - | .1129357 |
| Invasive HR (beats/min) | -.2 | -.28 | -.12 | 5.25e-07 | .0000438 | .4968159 |
| Serum triglycerides (mmol/l) | -.17 | -.24 | -.09 | .0000134 | .0014236 | .0045059 |
| PWV (m/s) | -.18 | -.26 | -.09 | .0000474 | .0006939 | .4500026 |
| HDL-cholesterol (mmol/l) | .18 | .1 | .27 | .0000537 | .0100088 | .3568551 |
| Manual DBP (mmHg) | -.17 | -.25 | -.08 | .0000735 | .0117573 | .9518756 |
| Night/day-ratio DBP | -.15 | -.23 | -.08 | .0001297 | .0001671 | .2094017 |
| Central DBP (mmHg) | -.15 | -.24 | -.07 | .000197 | .0275703 | .3742021 |
| FEV1 (L) | .21 | .1 | .31 | .0002345 | .0003782 | .3465481 |
| Resting VC (L) | .22 | .1 | .33 | .0002841 | .0011822 | .5153528 |
| Central SBP (mmHg) | -.15 | -.23 | -.07 | .000387 | .0500232 | .9166009 |
| Fasting blood glucose (mmol/l) | -.17 | -.27 | -.07 | .000729 | .0034434 | .4709649 |
| White coat effect DBP (mmHg) | .13 | .06 | .21 | .0008071 | .0032135 | .243476 |
| s´ at TDI (cm/s) | .13 | .05 | .21 | .0016635 | .0129005 | .0720136 |
| SDFV ratio | .13 | .05 | .21 | .0020879 | .0027362 | .9404823 |
| Night/day-ratio heart rate | -.12 | -.2 | -.04 | .0029618 | .0165846 | .1650529 |
| Manual SBP (mmHg) | -.12 | -.2 | -.04 | .0037786 | .1989613 | .2616363 |
| AIx | -.13 | -.21 | -.04 | .0039436 | .0067438 | .1816143 |
| Night/day-ratio SBP | -.11 | -.19 | -.04 | .0039926 | .0031999 | .0381101 |
| e´/a´-ratio at TDI | .12 | .04 | .19 | .0040376 | .015449 | .2307157 |
| Ambulatory SBP (mmHg) | -.12 | -.2 | -.04 | .0047943 | .2435959 | .3647082 |
| IM-GSM | .1 | .03 | .18 | .0087057 | .1903731 | .9480643 |
| Carotid diameter (mm) | -.13 | -.23 | -.03 | .0087363 | .074631 | .4181174 |
| e´ at TDI (cm/s) | .1 | .03 | .18 | .0089416 | .0413642 | .3028639 |
| Ambulatory DBP (mmHg) | -.11 | -.2 | -.03 | .0092311 | .1893038 | .1296295 |
| Invasive SBP (mmHg) | -.12 | -.21 | -.03 | .0115973 | .1228935 | .4992253 |
| SI (ml/BSA) | .1 | .02 | .18 | .0148909 | .0345744 | .2689021 |
| e/a-ratio at TDI | .09 | .01 | .17 | .0270637 | .1434431 | .3440402 |
| Invasive PP (mmHg) | -.1 | -.19 | -.01 | .0279404 | .1075776 | .0992078 |
| Invasive DBP (mmHg) | -.1 | -.19 | -.01 | .031763 | .3072031 | .7082931 |
| LF (normalized) (%) | .09 | .01 | .17 | .0337144 | .0513193 | .0686189 |
| pNN50 (%) | .09 | .01 | .18 | .0342462 | .0900191 | .2055522 |
| LA (mm) | -.09 | -.18 | -.01 | .0377579 | .8969989 | .7283968 |
| RI | .08 | 0 | .16 | .03868 | .0814317 | .3304406 |
| EIDV 5 µg (%) | .11 | .01 | .22 | .039157 | .282001 | .3541011 |
| White coat effect SBP (mmHg) | .08 | 0 | .16 | .0399868 | .0892663 | .0083685 |
| Resting VCO_2_ (ml/min) | -.1 | -.19 | 0 | .0429475 | .9648238 | .3950094 |
| LF/HF ratio | .08 | 0 | .17 | .0524184 | .105064 | .5025766 |
| RWT | -.08 | -.16 | 0 | .0539312 | .0305633 | .4564734 |
| Central PP (mmHg) | -.08 | -.16 | 0 | .0556723 | .3867825 | .3732791 |
| Brachial flow at rest  (ml/min/100 ml tissue) | -.09 | -.19 | 0 | .0564682 | .5173517 | .000278 |
| EIDV 10 µg (%) | .1 | 0 | .21 | .0572336 | .4195701 | .483199 |
| Transmitral A-wave (cm/s) | -.08 | -.17 | 0 | .0581418 | .2897874 | .1707306 |
| LVEDD (mm) | .09 | -.01 | .18 | .0671884 | .0045698 | .585731 |
| LVEDV (cm^3^) | .09 | -.01 | .18 | .0680586 | .0049554 | .6348581 |
| HRV-index (ms) | .09 | -.01 | .19 | .0760981 | .2918703 | .2720193 |
| SV/PP-ratio (ml/mmHg) | .08 | -.01 | .16 | .0801433 | .0744209 | .1745285 |
| HF (normalized) (%) | -.07 | -.15 | .01 | .0857731 | .1558537 | .0332949 |
| NN50 (ms) | .07 | -.01 | .16 | .0881798 | .1363745 | .2520652 |
| Resting energy expenditure (kcal) | -.08 | -.17 | .02 | .1141029 | .6261423 | .5339937 |
| Resting VO_2_ (ml/min) | -.08 | -.17 | .02 | .1179873 | .6323502 | .6820561 |
| LVESD (mm) | .07 | -.02 | .16 | .1203007 | .0383165 | .7104979 |
| EDV 50 µg (%) | .07 | -.02 | .16 | .1211521 | .3884597 | .7672588 |
| EDV 25 µg (%) | .07 | -.02 | .16 | .1410604 | .3179322 | .4327944 |
| LVESV (cm^3^) | .06 | -.02 | .15 | .1601087 | .05641 | .7803703 |
| LF maximum (Hz) | .06 | -.02 | .14 | .1623269 | .3396007 | .6621368 |
| Ambulatory HR (beats/min) | -.05 | -.13 | .02 | .18199 | .4818081 | .2278306 |
| HF maximum (Hz) | -.06 | -.14 | .03 | .1917375 | .4493958 | .736066 |
| E/e´-ratio at TDI | -.05 | -.14 | .03 | .2069677 | .4876007 | .9095241 |
| TPRI (dynes*s*cm^-5^*BSA) | -.05 | -.13 | .03 | .2096033 | .4528221 | .8086194 |
| FMD (%) | .05 | -.03 | .13 | .2273432 | .1982024 | .0625979 |
| Transmitral E-wave (cm/s) | .05 | -.03 | .14 | .2287259 | .2054923 | .8474787 |
| a´ at TDI (cm/s) | -.05 | -.14 | .03 | .2464223 | .2750255 | .6171021 |
| Resting RQ | -.05 | -.12 | .03 | .2591389 | .2817072 | .266414 |
| Total power (ms^2^) | .04 | -.03 | .12 | .2653199 | .2907196 | .0934242 |
| IMT (mm) | -.04 | -.12 | .04 | .293353 | .5873536 | .2316629 |
| RMSSD (ms) | .03 | -.04 | .11 | .3770151 | .4974008 | .0478876 |
| Brachial diameter (mm) | -.05 | -.17 | .07 | .4003724 | .8588903 | .4686047 |
| CI (L/min/BSA) | -.03 | -.11 | .05 | .4381923 | .5445797 | .285362 |
| Manual PP (mmHg) | -.03 | -.11 | .05 | .4809313 | .6788045 | .0132939 |
| PW (mm) | -.03 | -.11 | .05 | .4927208 | .8843933 | .7401347 |
| IVS (mm) | -.03 | -.11 | .05 | .4930002 | .9105178 | .5273526 |
| Serum cholesterol (mmol/l) | .02 | -.06 | .1 | .5638523 | .2588177 | .033495 |
| RHI | .02 | -.06 | .11 | .6232511 | .7240347 | .5588076 |
| Brachial flow increase (%) | .01 | -.07 | .1 | .7435925 | .8341077 | .0677722 |
| EF | -.01 | -.09 | .07 | .7542755 | .9855546 | .9790934 |
| LVMI (g/m^2.7^) | -.01 | -.09 | .07 | .8031265 | .6038083 | .4158435 |
| IVRT (ms) | -.01 | -.09 | .07 | .8274232 | .7095273 | .3827294 |
| Hemoglobin (g/l) | .01 | -.09 | .12 | .8283484 | .440771 | .7839005 |
| Triangular index (ms) | 0 | -.08 | .07 | .9079096 | .6577349 | .234421 |

PP=Pulse pressure, HR=Heart rate, PWV=Pulse wave velocity, HDL=High density lipoprotein, DBP=Diastolic blood pressure, FEV1=Forced expiratory volume at 1 s, VC=Vital capacity, SBP=Systolic blood pressure, TDI=Tissue Doppler imaging, SDFV ratio=Systolic to diastolic blood flow velocity ratio, AIx=Aortic augmentation index, IM-GSM=Echogenicity of the intima-media complex, SI=Stroke index, BSA=Body surface area, LF=Low frequency, LA=Left atrial diameter, RI=Reflectance index, EIDV=Endothelium-independent vasodilatation, VCO_2_=Carbon dioxin production, HF=High frequency, RWT=Relative wall thickness, A= Atrial contraction transmitral filling velocity, LVEDD=Left ventricular end-diastolic diameter, LVEDV=Left ventricular end-diastolic volume, HRV=Heart rate variability, SV/PP-ratio=Stroke volume to pulse pressure ratio, VO_2_=Oxygen consumption, LVESD=Left ventricular end-systolic diameter, EDV=Endothelium- dependent vasodilatation, LVESV=Left ventricular end-systolic volume, TPRI=Total peripheral resistance index, FMD=Flow-mediated dilatation, E=Early transmitral filling velocity, RQ=Respiratory quote, IMT=Intima-media thickness, CI=Cardiac index, PW=Posterior wall thickness, IVS=Intraventricular thickness, RHI=Reactive hyperemia index, EF=Ejection fraction, LVMI=Left ventricular mass index, IVRT=Isovolumetric relaxation time
